# Supplementary figures and images for: The Zooxanthellate Jellyfish Holobiont Cassiopea andromeda, a Source of Soluble Bioactive Compounds
Source: Mar Drugs. 2023 Apr 26;21(5):272. doi: 10.3390/md21050272 (PMC10222531; doi:10.3390/md21050272)

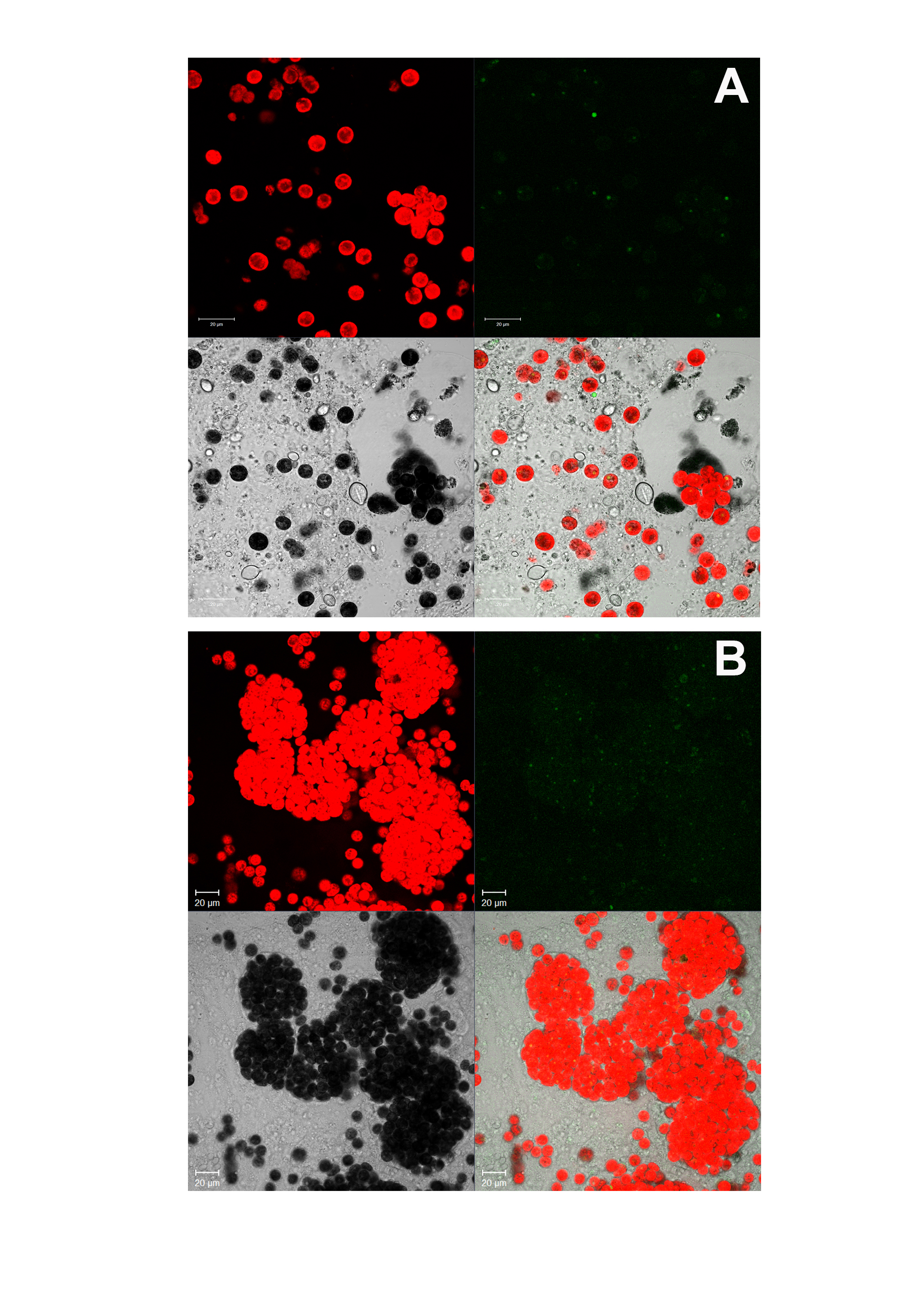

Supplement: Supplementary file 1 [file marinedrugs-21-00272-s001.zip › Figure S1.jpg]

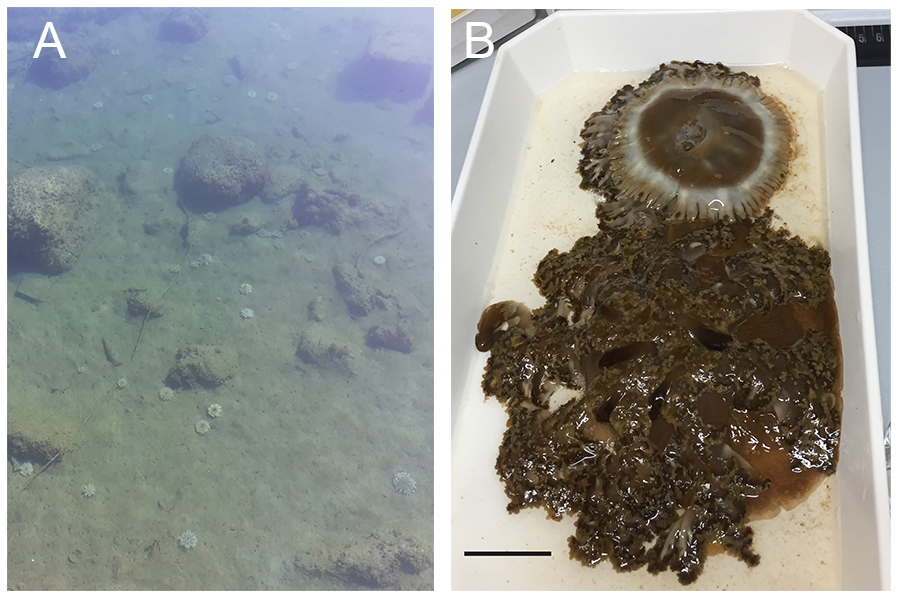

Supplement: Supplementary file 1 [file marinedrugs-21-00272-s001.zip › Figure S2.jpg]

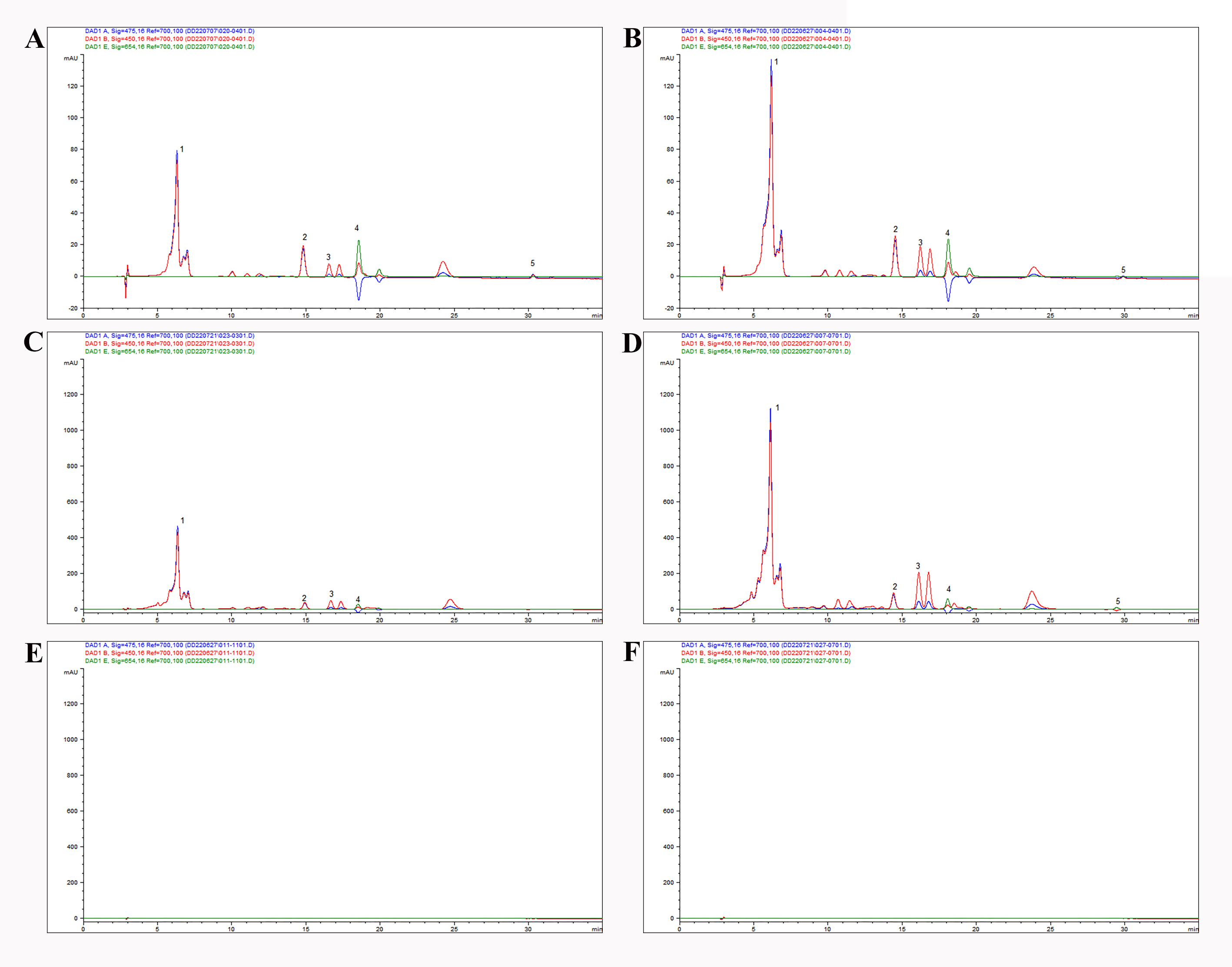

Supplement: Supplementary file 1 [file marinedrugs-21-00272-s001.zip › Figure S3.jpg]
